# Myelin mapping in patients with rheumatoid arthritis-related fatigue: a TBSS-MTR study of integrity

**DOI:** 10.1093/bjro/tzaf014

**Published:** 2025-05-24

**Authors:** Maryam Alhashim, Neil Basu, Alison Murray, Gordon Waiter

**Affiliations:** Medical Physics Department, Medical Imaging Services Canter, King Fahad Specialist Hospital, Dammam 31444, Saudi Arabia; Department of Radiology, College of Medicine, Imam Abdulrahman Bin Faisal University, Dammam 34212, Saudi Arabia; Institute of Infection, Immunity and Inflammation, University of Glasgow, Glasgow G12 8TA, United Kingdom; Aberdeen Biomedical Imaging Centre, University of Aberdeen, Aberdeen AB25 2ZD, United Kingdom; Aberdeen Biomedical Imaging Centre, University of Aberdeen, Aberdeen AB25 2ZD, United Kingdom

**Keywords:** rheumatoid arthritis, fatigue, magnetization transfer imaging, diffusion tensor imaging, tract-based spatial statistics

## Abstract

**Background:**

Rheumatoid arthritis (RA) patients frequently report fatigue, which notably diminishes their quality of life. Emerging research points to a correlation between inflammation-induced fatigue and brain structural alterations.

**Objectives:**

This study evaluates the variance in myelin integrity among patients with RA-related fatigue, investigating the potential of magnetization transfer ratio (MTR) as a biomarker, in comparison with healthy controls.

**Methods:**

A prospective cohort analysis was conducted comprised 60 RA patients with fatigue, categorized into active (*n* = 30) and non-active (*n* = 30) disease states, alongside 20 healthy controls (HC). A 3 Tesla MRI system was utilized to perform diffusion tensor imaging (DTI) and magnetization transfer imaging (MTI) sequences. MTR maps were generated using in-house MATLAB code and co-registered with DTI data using SPM8. These were then analyzed through tract-based spatial statistics (TBSS) with threshold-free cluster enhancement (TFCE) and corrected for multiple comparisons. MTR values were assessed using Randomize from the FSL toolkit, applying a general linear model (GLM) for voxel-wise analysis and TFCE for p-value generation, with family-wise error (FWE) control (*P* < .05) for multiple comparisons.

**Results:**

The RF group exhibited significantly lower myelin integrity (TFCE, *P* < .05) compared to HCs, particularly in the middle cerebellar peduncle and splenium of the corpus callosum, with no marked difference between active and non-active RA disease statuses. There is a discernible disparity in myelin integrity between RA patients with fatigue and healthy individuals, suggesting microstructural white matter alterations that are congruent with DTI findings.

**Conclusion:**

This study reveals that rheumatoid arthritis (RA) patients with fatigue exhibit significantly lower myelin integrity, particularly in the middle cerebellar peduncle and splenium of the corpus callosum, compared to healthy controls. Notably, this finding was consistent regardless of the active or non-active status of the RA disease, highlighting persistent white matter alterations in this patents cohort.

**Advances in knowledge:**

The research demonstrates that magnetization transfer ratio (MTR) imaging can effectively map microstructural changes in RA patients with fatigue, suggesting its potential as a biomarker for assessing white matter integrity in this condition. While it does not establish a direct causal relationship, it provides valuable insights into the role of MTR mapping in understanding brain alterations in patients with fatigue-related RA.

## Introduction

Fatigue is a major problem that is commonly reported by patients with rheumatic disorders such as rheumatoid arthritis (RA).^1^ It manifests as extreme tiredness, lack of motivation and mental strain that affect patients energy, memory, concentration and correlated with poor quality of life.^2^ According to Piecyk et al, RA may occur with a range of nonarticular central and peripheral neurologic conditions such as myelopathy, peripheral neuropathy, and compressive neuropathy.^3^ The literature suggesting a link between inflammatory related fatigue and structural and functional changes in brain is accumulating.^4–6^ The advances in neuroimaging techniques have contributed to the understanding of brain involvement in inflammatory condition.^6^ Several studies have used neuroimaging to address and investigate the relationship between a number of neural correlates and subjective measures of fatigue,^7^^,^^8^ suggesting that the brain plays a role in the symptoms underlying inflammatory-related fatigue disorders. One of the essential components that is responsible for neuronal communication is myelin, a dielectric protein, which forms a fatty sheath surrounding axons. The assessment and evaluation of myelin integrity is the key to achieve a complete understanding of the fatiguing process.^9^

Magnetization transfer imaging (MTI) is an advanced MRI technique that is sensitive to changes in the biochemical composition of tissues and hence provides a quantitative assessment of myelin integrity based on the exchange of magnetization between the relatively free water pool (ie mobile protons) and restricted protons bound to macromolecule (ie those contained within myelin). The relationship between these two quantities is measured by the magnetization transfer ratio (MTR);^10^ a dimensionless quantity computed from MTI image which varies as a function of specific tissues parameters and is summarized by the following model:


(1) $$\mathrm{MTR}\propto kf_{b}T_{1},$$

where *k* is a relaxation constant between myelin and other tissues, *f_b_* represents the fraction of protons in myelin and *T*_1_ is longitudinal relaxation time.^11^ Lower values of MTR reflect either (1) a lower capacity of myelin to exchange magnetization with surrounding molecules resulting in alterations in myelin integrity (ie, lower *f_b_*) or (2) structural changes following inflammation (ie, lower T1).^9^

Many studies have demonstrated the existence of correlations between fatigue and myelin changes in the brain, as determined using MTR, among patients with inflammatory and non-inflammatory disorders.^12–14^ In RA disease, where inflammation mainly attacks the joint, treatment pathways are limited due to the lack of understanding of the fatigue origin, it has not been investigated whether RA-related fatigue myelin integrity is similar to healthy controls.

Global voxel-wise MTR analysis using the tract-based spatial statistics (TBSS) technique offers some advantages in investigating fatigue, particularly in conditions such as RA where there is no gold standard alternative. As MTR is a powerful tool that is sensitive to changes in both myelin and axonal density, it has been widely used in the assessment of WM diseases such as multiple scleroses. For example, a significantly lower whole brain MTR measures was associated with cognitive impairment which is linked to the mental fatigue as it interferes with the cognitive performance of patients in the very early stages of multiple sclerosis.^13^

Our work aims to investigate the potential of a MTI derived measurement of myelin integrity, MTR, as a potential biomarker of microstructural changes. We focus on a well-characterized group of patients with rheumatoid arthritis (RA), a condition in which fatigue is a prominent and debilitating symptom. By studying this cohort, we seek to:

- Assess differences in myelin integrity between patients with active and non-active RA, reflecting varying levels of disease burden that may influence fatigue.
- We chose to include a healthy control group to establish a baseline reference for brain myelin integrity, allowing us to observe how MTR maps appear in the absence of both disease and fatigue.
- Consolidate results of WM integrity from MTR with diffusion weighted tensor images (DTI) metrics, providing a broader perspective on white matter integrity.
- Compare myelin integrity in RA patients with that of healthy controls, to identify any disease-related microstructural alterations.

We have performed the first voxel-wise MTR TBSS analysis is to investigate the relationship between alterations in myelin integrity and the experience of fatigue in cohorts of rheumatic patients as well as between fatigue RA and healthy controls. Considering the existing literature, we hypothesize that there is a link between changes in myelin integrity and RA-related fatigue.

## Methods

### Demographics and clinical data

This prospective study included 60 consecutive patients with a diagnosis of RA (*n* = 60), having been classified according to the 2010 American College of Rheumatology/European League Against Rheumatism (ACR/EULAR) criteria,^15^ were recruited consecutively from secondary care rheumatology clinics in NHS Grampian before being grouped into active (*n* = 30) and non-active (*n* = 30) fatigue cohorts. Disease activity was assessed using DAS28 scoring system; disease activity score ≥3.3 to be considered as active disease.^16^ Fatigue was assessed in the RA group by completion of a self-report fatigue questionnaire, which was reported as clinically relevant when scoring more than 3 on the Chalder fatigue binary scale (CFS) and having lasted for over 3 months.^17^ In addition, 20 healthy controls (HC) completed the CFS questionnaire as well and were recruited as comparators for the study. All participants underwent a full clinical assessment except HC group. With regards to safety participants were contacted by phone to confirm if they had the following: (1) contraindications to MRI scanning (eg, pacemakers, artificial heart valves, artificial joints, nerve stimulators, artificial eyes, intrauterine devices) and (2) self-reported claustrophobia. In terms of study confounds, patients were excluded if they had: (1) left-handedness, (2) recent use of beta-blockers, or (3) hypertension. Additional exclusion criteria included: (4) other autoimmune or inflammatory conditions (eg, systemic lupus erythematosus, multiple sclerosis), (5) neurological or psychiatric disorders (eg, stroke, major depression, cognitive impairment), (6) history of traumatic brain injury, (7) current use of medications affecting central nervous system function (eg, corticosteroids, antidepressants), (8) chronic fatigue syndrome or fibromyalgia, (9) diagnosed sleep disorders, and (10) contraindications to MRI (eg, metal implants, claustrophobia). Prior to the study, each participant provided written informed consent after the study protocol was fully explained to him or her. Inclusion criteria for HC were age between 18 and 60 years, and no inflammatory history or fatigue. The North of Scotland Research Ethics Committee (NoSREC) approved the RA study and College Ethics Review Board (CERB) approved the healthy control study. After receiving all relevant ethical approvals, the study was conducted in accordance with the principles of the declaration of Helsinki 1964 and later revisions.

### MRI acquisition

All attending participants were scanned for the brain protocol with a SENSE-8-channel phased-array head coil in a 3 T Philips Achieva X-series, Netherlands, scanner.^18^ All structural T1 images were first reviewed by two consultant clinical radiologists, each with over 15 years of experience, to confirm that all participants—including healthy controls—were free from neurological abnormalities unrelated to RA fatigue (eg, lesions, atrophy, infarcts), ensuring suitability for inclusion. The radiologists were blinded to participants’ fatigue status to ensure an unbiased assessment. Following this, the T1 images were used for anatomical co-registration to facilitate accurate alignment of the quantitative MTR and DTI maps, tissue segmentation and masking, and spatial normalization to a standard template space (eg, Montreal Neurological Institute standard space MNI). This transformation was then applied to the MTR maps to allow for group-level comparisons.

#### High-resolution T1-weighted images

A 3D gradient-echo fast field echo (FFE) T1-weighted structural scan of the brain was obtained in 5 minutes and 35 seconds as a series of 160 sagittal slices with the following parameters: repetition time (TR) = 8.2 s, echo time (TE) = 3.8 ms, flip angle = 8˚, matrix size = 240 × 240 × 160, field of view (FOV) = 240 × 240 mm^2^, voxel size = 1.0 × 1.0 × 1.0 mm^3^.

#### DTI images

Whole brain diffusion weighted tensor images were recorded along 16 gradient directions (*b* = 800 s/mm^2^, number of excitations = 2) together with one un-weighted (*b* = 0) image with 17 volumes in total. A spin echo (SE) DTI images were obtained in a 4 min and 28 s as a series of 66 axial slices. Each image was acquired using a single-shot spin echo planar imaging (EPI) sequence with the following parameters: TR = 7151 ms, TE = 55 ms, flip angle = 90˚ slice thickness = 2.0 mm with no gap, FOV = 224 × 224 mm^2^, voxel size = 2 × 2 × 2 mm^3^, matrix size =224 × 224 × 132.

#### MTI images

MTI were acquired using 3D FFE technique in 8 min and 9 s as a series of 70 over-contiguous axial slices with the following parameters: TR/TE (ms) = 85/2.1, FOV = 224 × 168 mm^2^, voxel size 2 × 2 × 2 mm^3^, flip angle = 18˚, matrix size = 224 × 168 x 140 with bandwidth of 383 Hz.

### Analysis

Image data analysis was subsequently performed by one physicist, with a PhD and over 5 years of experience, using in-house MATLAB-written code (MATLAB R2008a; MathWorks, Natick, MA) as detailed below.

### Myelin integrity evaluation

MTR maps of the myelin integrity were computed using code written in MATLAB (R2008a),^19^ by calculating the percentage change between saturated ($M_{s}$) and unsaturated ($M_{o}$) images on a voxel-by-voxel basis according to the following formula (Price et al, 2014):


(2) $$\mathrm{MTR}=[M_{o}-\frac{M_{s}}{M_{o}}]\times \mathrm{percent}\;\mathrm{unit}.$$

**Figure 1.**
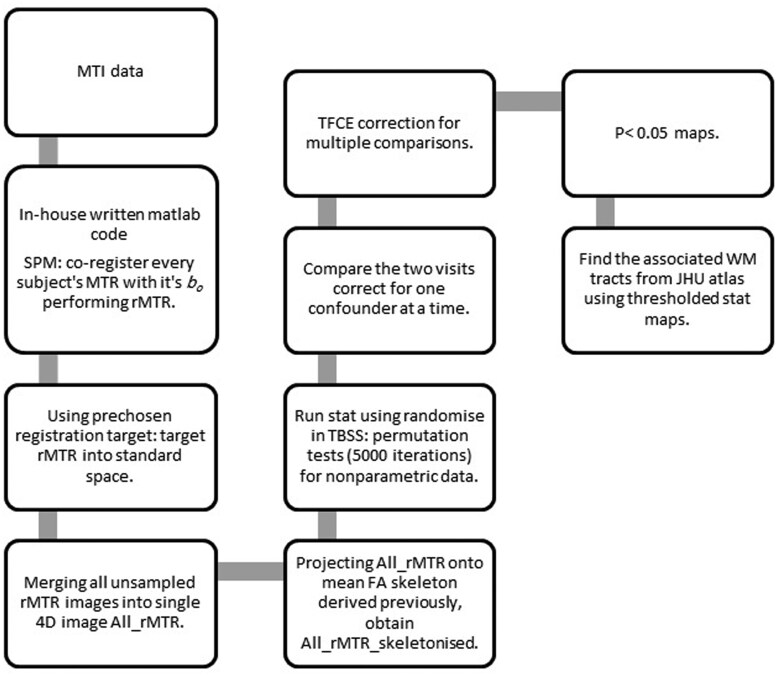
Skeletonization process of MTR maps. The figure shows the skeletonization of MTR maps using TBSS. MTR data were aligned to FA maps, normalized to Montreal Neurological Institute standard space MNI152 space, and projected onto a mean FA skeleton. Thresholding and quality checks ensured accuracy at each step.

Our method in combining DTI with MTR in a voxel-based TBSS analysis was first proposed by Bodini et al.^20^ Pre-processing of the MTR data started with alignment and registration to their corresponding fractional anisotropy (FA) images, derived from DTI scans to eliminate spatial distortion caused by head motion, using the Statistical Parametric Mapping 8 (SPM8) software.^21^ FA maps were generated using DTIfit, tool provided by FSL, a comprehensive library of analysis tools for DTI data.^22^ Voxel-wise statistical analysis of the FA maps was performed using TBSS, more details may be found in several publications.^23^^,^^24^ In brief all subjects FA and MTR maps were aligned, in a separate process, to FMRIB58_FA target image template before normalization using a non-liner transformation, to bring all subjects into a standard space; 1 × 1 × 1 mm^3^ Montreal Neurological Institute standard space (MNI152).^25^^,^^26^ After that the registered/normalized FA maps were all merged into a single 4-D image before being averaged and thinned to create a cross-subject mean FA image which is fed into the skeletonization program to generate a mean FA skeleton representing the centre of all tracts common to the group. The skeleton was then thresholded to a value of 0.2 to reduce inter-subject variability and partial volumes from grey matter with the aligned/normalized FA volumes of each subject being projected onto this skeleton with the aid of a distance map derived from it. A 4D image file of the projected skeletonized FA data was created and fed into voxel-wise cross-subject statistics. Finally skeletonized MTR maps were produced using the same projection as for FA process of transformed MTR onto a common FA tract skeleton. Consistent with best practice the resulting data was visually examined and verified after each processing step (Figure 1).

### MTR voxel-wise statistical analysis

The projected skeletonized MTR data were analysed using randomize, a non-parametric inference and permutation-based tool kit provided with FSL.^27^ Randomize uses the general linear model (GLM) to model data creating a voxel-wise test statistic images, before applying a threshold free cluster enhancement test (TFCE-based tests) to produce sets of *P*-value images. TFCE enhances the sensitivity of the analysis by using clusters to find significant areas in MTR data hence it is cluster-like structures are enhanced, but the image remains fundamentally voxel-wise. The results are controlled for multiple comparisons by using the family wise error (FWE) method (*P* < .05). The John Hopkins University DTI-based white-matter atlas, available through the Atlas query tool provided by FSL, was used to identify fibre tracts of interest.^28^

### Between-group imaging analysis

To test between-group differences, a two-group difference model adjusted for covariates was used. It expands the two-sample unpaired *t*-test to enable the inclusion of regressors in the model as covariates to be adjusted for. The inference of interest is whether the group difference remains after adjusting for covariates of interest. Corresponding matrix design and contrast matrices were fed into randomise with 5000 permutations per comparison. Again TFCE was used to produce P-maps results being reported at *P* < .05 before corrected for multiple comparisons using FWE. Finally, fibre tracts where significant differences were obtained were identified using Cluster, a program provided by FSL.^29^

## Results

### Demographic and clinical statistics

All data in Table 1 are presented in the format mean (standard deviation). The mean age of the RF and HC groups didn’t significantly differ from each other (*t* = 2.02, df = 78, *P*-value = 0.05). The mean of CFS score was statistically higher in RF at the 0.05 level of significance compared with HC, as expected (*t* = 20, df = 78, *P*-value = 0.0001). When comparing ACT to NON in the RF sub-groups we found that no significant difference in gender (Table 2), age (*t* = 2.2, df =58, *P*-value = 0.03), fatigue score (diff= 1.1, *P*-value = 0.2) or depression (diff = 1.3, *P*-value= 0.6). As expected, the level of daily pain intensity was significantly higher in ACT than NON (*t* = 4.078, df = 58, *P*-value =0.0001). Similarly, disease activity was significantly higher in ACT (*t* = 6.8, df = 58, *P*-value =0.0001). Sleep (*t* = 2.0109, df = 58, *P*-value =0.05) did not significantly differ between the ACT and NON groups.

**Table 1.** Demographic characteristics of cross-sectional subset, Chalder fatigue score (CFS), Hospital Anxiety and Depression Scale (HADS), disease activity score (DAS)28.

	RF group					
	ACT	NON	RF	HC	*t*(df)	*P*
Male:female	7:23	7:23	14:46	15:05	–	–
Age (years), mean (SD)	51.5 (12.4)	57.8 (9.2)	54.7 (11.4)	48.9 (10.3)	2 (78)	0.05
CFS,^a^ mean (SD)	9.7 (1.4)	8.6 (1.7)	9.2 (1.6)	1 (1.5)	20 (78)	<0.0001
Depression,^b^ mean (SD)	7.9 (3.8)	6.6 (4.4)	7.3 (4)	NA	–	–
Pain,^c^ mean (SD)	4.9 (2)	2.8 (2)	3.9 (2.3)	NA	–	–
DAS28,^d^ mean (SD)	4.5 (1.1)	2.8 (0.9)	3.6 (1.3)	NA	–	–
Sleep,^e^ mean (SD)	17.5 (5)	14.8 (5.7)	16.15 (5.5)	NA	–	–

a CFS; Chalder fatigue score from 0 to 10 if total ≥ 4 considered fatigue (Chalder et al, 1993).

b Depression; Hospital Anxiety and Depression Scale (HADS) Depression score ≥8 (Covic et al, 2012).

c Pain score; a numerical rating scale from 0 to 8 with scoring to answer the question “How severe is your pain right now?” (Bardwell, Nicassio, Weisman, Gevirtz, & Bazzo, 2002).

d DAS28; disease activity score ≥3.3 to be considered as active disease (VAN DE PUTTE & VAN RIEL, 1995).

e Sleep score; based on Jenkins’ sleep scale (Jenkins, Stanton, Niemcryk, & Rose, 1988).

**Table 2.** Summary of the voxel-wise comparison cross-sectional study by white matter tract, labels are identified using Johns Hopkins University (JHU) Diffusion tensor imaging (DTI)-based white-matter atlases.

White matter tracts	FA	MTR
Middle cerebellar peduncle	–	–
Pontine crossing tract (a part of MCP)	–	–
Genu of corpus callosum	–	0
Body of corpus callosum	–	0
Splenium of corpus callosum	–	–
Fornix (column and body of fornix)	–	–
Corticospinal tract R	–	–
Corticospinal tract L	–	–
Medial lemniscus R	–	–
Medial lemniscus L	–	–
Inferior cerebellar peduncle R	–	–
Inferior cerebellar peduncle L	–	–
Superior cerebellar peduncle R	–	–
Superior cerebellar peduncle L	–	–
Cerebral peduncle R	–	–
Cerebral peduncle L	–	–
Anterior limb of internal capsule R	–	0
Anterior limb of internal capsule L	–	0
Posterior limb of internal capsule R	–	–
Posterior limb of internal capsule L	–	–
Retrolenticular part of internal capsule R	–	–
Retrolenticular part of internal capsule L	–	–
Anterior corona radiata R	–	0
Anterior corona radiata L	–	0
Superior corona radiata R	–	0
Superior corona radiata L	–	0
Posterior corona radiata R	–	0
Posterior corona radiata L	–	–
Posterior thalamic radiation (include optic radiation) R	–	–
Posterior thalamic radiation (include optic radiation) L	–	–
Sagittal stratum (include inferior longitidinal fasciculus and inferior fronto-occipital fasciculus) R	–	–
Sagittal stratum (include inferior longitidinal fasciculus and inferior fronto-occipital fasciculus) L	–	–
External capsule R	–	–
External capsule L	–	–

– Lower values, 0 No change in values.

### Myelin integrity changes

#### Active disease RA vs non-active comparison

**Figure 2.**
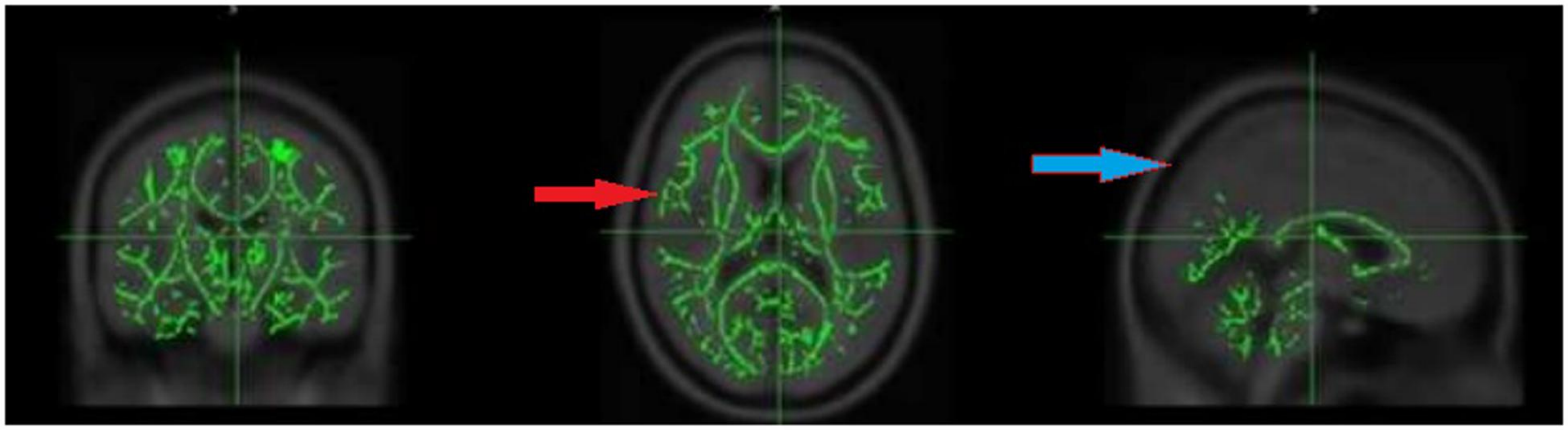
Group differences in MTR showing no significant differences in MTR values active RA group compared to non-active RA group. P maps are calculated at *P *< .05, family-wise error (FEW) corrected, and overlaid on the mean MTR skeleton (green),—red arrow, thresholded between 0.2 and 0.7 and the Montreal Neurological Institute standard space MNI125 T1 1 mm template,—blue arrow, using fsl open source software.

After controlling for the effects of depression and sleep, there were no significant between-group differences in MTR maps in any region of the brain after FWE correction (Figure 2).

#### Fatigue RA vs healthy controls

**Figure 3.**
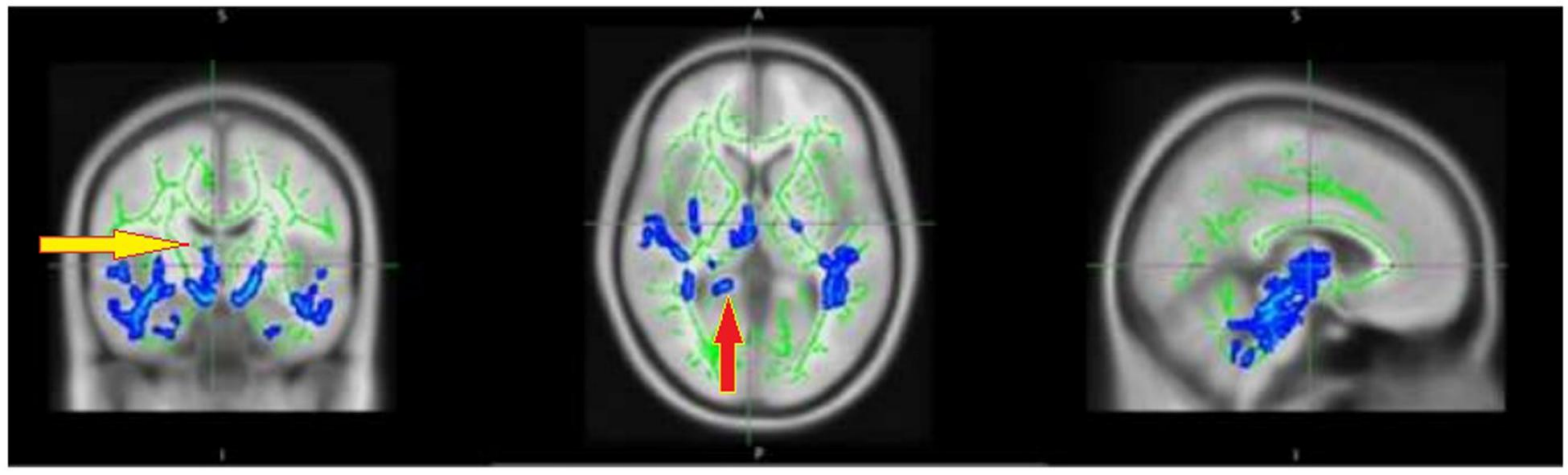
Cross sectional changes in MTR showing a significant lower MTR values in fatigue RA group compared to healthy controls. Significant voxels are shown in dark blue, for example Splenium of corpus callosum, red arrow, fornix, yellow arrow. P maps are displayed at *P *< .05, family-wise error (FEW) corrected, and overlaid on the mean MTR skeleton (green), thresholded between 0.2 and 0.7 and the Montreal Neurological Institute standard space MNI125 T1 1 mm template using fsl open source software.

There was a significant and highly diffuse lower value of myelin integrity in RF group in several areas compared to healthy controls. These tracts include the: middle cerebellar peduncle, pontine crossing tract, Splenium of corpus callosum, fornix, corticospinal tract, medial lemniscus, inferior cerebellar peduncle, superior cerebellar peduncle, posterior limb of internal capsule, retrolenticular part of the internal capsule bilaterally, left posterior corona radiate, posterior thalamic radiation, sagittal stratum (including the inferior longitudinal fasciculus and inferior fronto-occipital fasciculus), external capsule bilaterally and right cingulate gyrus, left superior longitudinal fasciculus, left uncinate fasciculus and right tapetum (Figure 3).

#### Comparing FA and MTR results

**Figure 4.**
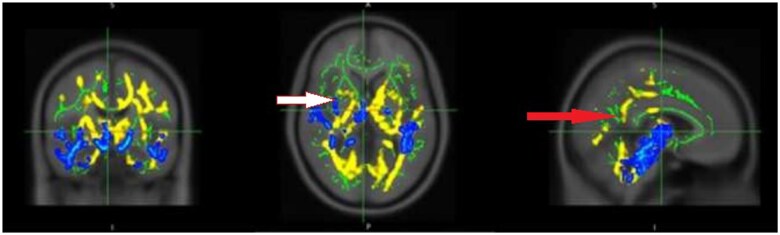
Significant decreases in MTR values (blue) overlaid on the decreases in FA values (yellow) found in the RA fatigue group compared to healthy controls, cingulum, red arrow, uncinate fasciculus, white arrow. P maps are displayed at *P* < .05, family-wise error (FEW) corrected, and overlaid on the mean MTR skeleton (green), thresholded between 0.2 and 0.7 and the Montreal Neurological Institute standard space MNI125 T1 1 mm template using FSL open source software.

When MTR significant maps were overlaid on the corresponding FA maps MTR showed more focally diffused WM tracts (Figure 4). These tracts tend to be localized more to the right hemisphere of the brain specifically in the cingulum (cingulate gyrus), fornix, uncinate fasciculus, and tapetum (Table 3).

**Table 3.** Cont. summary of the fractional anisotropy (FA) and magnetization transfer ratio (MTR) voxel-wise comparison.

White matter tracts	FA	MTR
Cingulum (cingulate gyrus) R	–	–
Cingulum (cingulate gyrus) L	–	0
Cingulum (hippocampus) R	–	0
Cingulum (hippocampus) L	–	0
Fornix (cres)/Stria terminalis R	–	–
Fornix (cres)/Stria terminalis L	–	0
Superior longitudinal fasciculus R	–	0
Superior longitudinal fasciculus L	–	–
Superior fronto-occipital fasciculus (could be a part of anterior internal capsule) R	–	0
Superior fronto-occipital fasciculus (could be a part of anterior internal capsule) L	–	0
Uncinate fasciculus R	0	–
Uncinate fasciculus L	–	–
Tapetum R	–	–
Tapetum L	–	0

– Lower values, 0 No change in values.

## Discussion

In rheumatoid arthritis patients with fatigue manifestations, and in most cases, conventional MRI sequences cannot detect neural abnormality.

We used a voxel-based analysis of MTR values to explore the myelin integrity changes in a unique cohort study of patients with RA-related fatigue. This work aimed to simplify the challenge of differentiating microstructural integrity changes in RA-related fatigue compared to HC, and changes caused by inflammation by comparing RA active vs non active patients. When RA patients were compared to the HC, RA patients showed a significant lower (TFCE, *P* < .05) in MTR along WM tracts, such as the middle cerebellar peduncle, pontine crossing tract, splenium of corpus callosum, fornix, corticospinal tract, medial lemniscus, inferior cerebellar peduncle, superior cerebellar peduncle, posterior limb of internal capsule, retrolenticular part of internal capsule bilaterally, left posterior corona radiate, posterior thalamic radiation, sagittal stratum (include inferior longitidinal fasciculus and inferior fronto-occipital fasciculus), external capsule bilaterally and right cingulate gyrus, left superior longitudinal fasciculus, left uncinate fasciculus and right tapetum compared to healthy controls (Tables 2 and 3). These differences are not fatigue specific, rather it might result from altered motor control and prolonged pain complications; however, it is consistent with Phukan et al, in which prolonged inflammation could affect the central nervous system leading to axonal and myelin content alteration of WM fibre tracts.^30^

We demonstrated that some of these diffuse changes were mirrored by the DTI results reported in Tables 2 and 3 mainly in the middle cerebellar peduncle, pontine crossing tract, splenium of corpus callosum, fornix, corticospinal tract bilaterally, medial lemniscus bilaterally, inferior and superior cerebellar peduncle bilaterally, cerebral peduncle bilaterally, posterior limb of internal capsule bilaterally, retrolenticular part of internal capsule bilaterally, left posterior corona radiate, left posterior thalamic radiation, sagittal stratum (include inferior longitidinal fasciculus and inferior fronto-occipital fasciculus) bilaterally, external capsule bilaterally, right cingulum, right fornix, left superior longitudinal fasciculus, left uncinate fasciculus and right tapetum.

Only one study has previously used TBSS analysis techniques with MTR measures of the brain to identify microstructural changes in the case of patients with multiple sclerosis at baseline and a 1-year follow-up.^20^ Results from this study support the hypothesis that RA-related fatigue is linked to alterations in WM integrity.

However, as RA is a medical condition that is further complicated by many factors besides fatigue, the changes in WM integrity can relate to many other factors, for example, inflammation, drugs, depression, sleep, etc. and cannot directly related to fatigue.

There were no significant differences between the active and non-active RA groups anywhere in the brain suggesting that disease activity cannot explain the fatigue experienced in rheumatoid arthritis especially after treatment. This result is consistent with findings in other previous studies.^1^^,^^31^^,^^32^

Our study aligns with Gonie et al's findings that brain structural metrics offer valuable insights into fatigue in RA patients.^33^ While Gonie et al utilized surface-based metrics such as the curvature of the superior temporal sulcus and focused on predicting fatigue improvement over time, our research centered on voxel-based MTR mapping to identify microstructural white matter alterations associated with fatigue.

Notably, Gonie et al reported a predictive accuracy of 67.9% using structural MRI (sMRI) features, emphasizing the utility of brain metrics over clinical measures. Similarly, our findings demonstrate that MTR mapping can detect significant differences in white matter integrity between RA patients with fatigue and healthy controls. Unlike Gonie et al, who focused on longitudinal fatigue changes and classification using artificial intelligence, our work provides a cross-sectional view of white matter changes using a complementary imaging modality.

Both studies underscore the importance of brain imaging as a potential tool to enhance the understanding and management of RA-related fatigue. However, while Gonie et al highlight predictive modeling for fatigue improvement, our study emphasizes the utility of MTR as a biomarker for microstructural integrity, particularly in regions like the middle cerebellar peduncle and splenium of the corpus callosum. This complementary perspective broadens the scope of neuroimaging biomarkers.

Liu et al identified reduced FA in major white matter tracts, such as the forceps minor and anterior thalamic radiation, in RA patients without specific focus on fatigue, linking these changes to pain and disease activity. In contrast, our study highlights microstructural alterations using MTR mapping in RA patients with fatigue, particularly in the middle cerebellar peduncle and splenium of the corpus callosum, independent of disease activity.^34^

While Liu et al focused on FA and pain, our use of MTR emphasizes myelin integrity, suggesting fatigue-related white matter changes may involve distinct neurobiological pathways. Together, these studies demonstrate the value of combining biomarkers to understand white matter alterations in RA.

This is the first cross-sectional myelin integrity study investigating fatigue among patients with RA and healthy controls. We used voxel-based TBSS analysis and the RA study groups were age and gender matched to decrease possible individual variability.

## Conclusion

We have established a healthy MTR TBSS baseline maps, providing a valuable reference for future studies on myelin integrity. Our findings highlight the potential of MTR as a biomarker for microstructural changes in RA-related fatigue. We recommend further investigation into RA patients without fatigue to better isolate and understand the specific impact of fatigue on brain microstructure.

### Limitations and future direction

Our study acknowledges certain limitations. The cross-sectional design, although effective in identifying associations, cannot establish causality between myelin integrity changes and RA-related fatigue. Additionally, our sample size, though adequate for detecting significant differences, may limit the generalizability of our findings. Future longitudinal studies with larger cohorts to study RA fatigue group versus RA without fatigue to stratify the fatigue dimension are necessary to validate our results and explore the temporal relationship between myelin changes and fatigue progression in RA. Furthermore, while we controlled for various confounding factors, other unmeasured variables, such as medication history and lifestyle factors, might have influenced the results.
